# AGING IN ENVIRONMENTALLY RISKY AREAS: FINDINGS FROM A NATIONALLY REPRESENTATIVE SURVEY

**DOI:** 10.1093/geroni/igad104.1886

**Published:** 2023-12-21

**Authors:** Alexis Merdjanoff, Katie Lynch

**Affiliations:** New York University, New York City, New York, United States; New York University, New York City, New York, United States

## Abstract

The Aging in Risky Environmental Areas (AREA) Study is a novel cross-sectional dataset of community-dwelling adults in the U.S. ages 50 and older. The sample of 1,504 participants was collected in November 2022 and is nationally representative. The survey examines several topics related to aging during the climate emergency including, recent climate-related disaster exposure; perspectives on climate adaptation and mitigation strategies; disaster resilience; and, various measures of healthy aging. There is a misconception that a majority of older adults do not believe in climate change or are unwilling to make behavioral changes to decrease their carbon footprint, despite their vulnerability to climate-related disasters. Results from the AREA Study demonstrate that a majority of older adults (50+) are willing to make “a lot” or “some” changes to the way they live or work in order to reduce the impact of climate change and that there are no statistically significant differences among age groups (50-64; 65-74; 75+). Instead, factors such as region, political affiliation, prior disaster exposure, and place attachment are significant predictors of willingness to engage in adaptation strategies. Findings suggest that an overwhelming majority of older adults (74%) believe that climate change is happening and are willing to be active participants in efforts to reduce negative impacts.

